# The Boston lymphatic center’s early experience with lymph node transplantation to the upper extremity

**DOI:** 10.20517/2347-9264.2022.77

**Published:** 2022-10-13

**Authors:** Rosie Friedman, Monica Morgenstern, Valeria P. Bustos, Aaron Fleishman, Kathy Shillue, Leo L. Tsai, Jonathan F. Critchlow, Dhruv Singhal

**Affiliations:** 1 Division of Plastic and Reconstructive Surgery, Department of Surgery, Beth Israel Deaconess Medical Center, Harvard Medical School, Boston, MA 02215, USA.; 2 Division of Magnetic Resonance Imaging, Department of Radiology, Beth Israel Deaconess Medical Center, Harvard Medical School, Boston, MA 02215, USA.; 3 Department of Surgery, Beth Israel Deaconess Medical Center, Harvard Medical School, Boston, MA 02215, USA.

**Keywords:** Lymphedema, vascularized lymph node transplant, lymphatic surgery

## Abstract

**Aim:**

Although vascularized lymph node transplantation (VLNT) has gained recognition as an effective treatment option for lymphedema, no consensus on the timing of transplant with other lymphatic procedures has been established. The aim of this study is to describe our institutional experience with VLNT, including our staged approach and report postoperative outcomes.

**Methods:**

A retrospective review of patients who underwent VLNT for upper extremity lymphedema from May 2017 to April 2022 was conducted. Patients were divided into fat- or fluid-dominant phenotypes based on preoperative workup. Patients with a minimum of 12-month follow-up were included. Records were reviewed for demographic, intraoperative, and surveillance data.

**Results:**

Twenty-three patients underwent VLNT of the upper extremity during the study period, of which eighteen met the study criteria. Nine patients had fluid-dominant disease and nine patients had fat-dominant disease and had undergone prior debulking at our institution. Fluid-dominant patients demonstrated slight reductions in limb volume and hours in compression, and improvement in quality-of-life scores at twelve months. Fat-dominant patients who underwent prior debulking had a slight increase in limb volume without a change in hours of compression, and demonstrated improvements in quality-of-life scores in nearly all subdomains. Overall, 17% of patients discontinued compression therapy entirely. Improvement in extremity edema was present in 83% of postoperative MRIs.

**Conclusion:**

VLNT had varying effects on limb measurements while reliably improving quality-of-life and allowing for the potential of discontinuing compression. Utilizing a staged approach wherein debulking is performed upfront may be particularly beneficial for patients with fat-dominant disease.

## INTRODUCTION

Upper extremity lymphedema is a debilitating and progressive disease with a substantial impact on patient quality of life^[1–4]^. Conservative therapies such as decongestive physiotherapy and compression garments are aimed at the palliation of symptoms and prevention of disease progression, but in certain cases, surgical interventions are deemed necessary. An evolving body of evidence demonstrates the beneficial effects of vascularized lymph node transplant (VLNT) on patient quality of life, occurrence of infection, and limb volumes in patients with extremity lymphedema^[5–8]^. Given its efficacy, VLNT has become a mainstay of treatment for lymphedema and expanding recognition has even led to the creation of a medical policy for insurance coverage for lymphatic surgery, including VLNT^[9]^. As VLNT has become increasingly adopted by lymphatic centers, programs have developed a staged approach in which VLNT and debulking lipectomy are performed sequentially in efforts to optimize patient outcomes^[10–15]^. However, because VLNT and debulking greatly differ in their underlying mechanisms and postoperative requirements for compression therapy, the timing and relation of these procedures require careful consideration. To date, a unified consensus has yet to be established on time intervals or the sequence of staged VLNT in relation to debulking lipectomy^[16]^.

Multiple studies have described a staged approach to treat upper extremity lymphedema. Schaverien *et al*. suggested performing suction-assisted liposuction after physiologic operations to remove excess fatty tissue that VLNT was unable to address^[12]^. In a similar manner, Nicoli *et al*. performed laser-assisted liposuction one to three months after VLNT^[11]^. Similarly, Agko *et al*. performed liposuction six to eight months after VLNT^[13]^. Cheng *et al*. proposed using liposuction after VLNT for patients with lipodystrophy in the proximal limb to decrease the burden of excess fluid on the lymph node flap^[10]^. Conversely, Cook *et al*. performed VLNT ten weeks after debulking lipectomy^[15]^. Similarly, Granzow *et al*. reported first performing debulking followed by VLNT six to twelve months later to improve functional lymphatic drainage, reduce ongoing fluid accumulation, and decrease the need for compression therapy^[14]^. Interestingly, these procedures have also been used simultaneously to treat upper extremity lymphedema^[17]^.

At our multi-disciplinary lymphatic center, we have implemented a VLNT program and standardized treatment approach based on patient classification as fat- or fluid-dominant lymphedema phenotype^[18–20]^. At our center, a debulking lipectomy is consistently performed upfront for patients with a fat-dominant phenotype, followed by a staged VLNT one to two years postoperatively. Patients with a fluid-dominant phenotype are offered VLNT without undergoing a prior debulking procedure. In the current study, we aim to describe our institutional experience with VLNT for the treatment of upper extremity lymphedema and report our postoperative outcomes, including limb volume measurements in the setting of hours of compression therapy per week, radiographic changes, and quality of life. In addition, we describe our management protocol when a combination of VLNT and debulking lipectomy is required for patients with a fat-dominant phenotype.

## METHODS

### Study design, setting, and population

An observational study was conducted at the Boston Lymphatic Center/Beth Israel Deaconess Medical Center. Institutional review board approval was obtained for this study (Protocol #2022P000092). A review of a prospectively maintained REDCap Quality Improvement Database^[21]^ and a medical review were performed. Patients who underwent vascularized omental lymph node transplant for the treatment of upper extremity lymphedema from May 2017 to April 2022 were identified. Patients were included if they had preoperative measurements, a minimum of 12 months of follow-up, and were treated as per our current algorithm in which patients with fat-dominant diseases underwent debulking lipectomy prior to VLNT. Patient demographics, lymphedema characteristics, intraoperative variables, and surveillance data were extracted for analysis. Baseline characteristics were summarized using means and standard deviations or medians and interquartile ranges (IQR) for continuous data and counts and percentages for categorical data. Descriptive data analysis was performed using R version 4.1.3 (R Foundation for Statistical Computing, Vienna, Austria).

### Preoperative evaluation and identification of surgical candidates

Our center’s approach and evaluation of a patient with lymphedema have been previously described^[18]^. Determination of lymphedema phenotype (fluid- versus fat-dominant) was performed by an attending radiologist (Tsai LL) as part of our standardized algorithm for evaluation of patients presenting to our center^[19,20]^. A T2-weighted short-T1 inversion recovery (STIR) image and fat-specific Dixon image were obtained and utilized to grade the proportion of fatty and fluid tissue in the affected limb. Patients with fat-dominant disease who underwent prior debulking were evaluated for VLNT at least one year post debulking lipectomy with stabilized limb volume. Those with a fluid-dominant phenotype were considered for VLNT alone.

### Surgical technique

This surgical procedure was performed collaboratively with plastic surgery (Singhal D) and general surgery (Critchlow JF) teams at our institution. Operative notes were reviewed to determine intraoperative details, including the microvascular anastomotic technique.

### Intraoperative duplex ultrasonography

**Figure 1.**
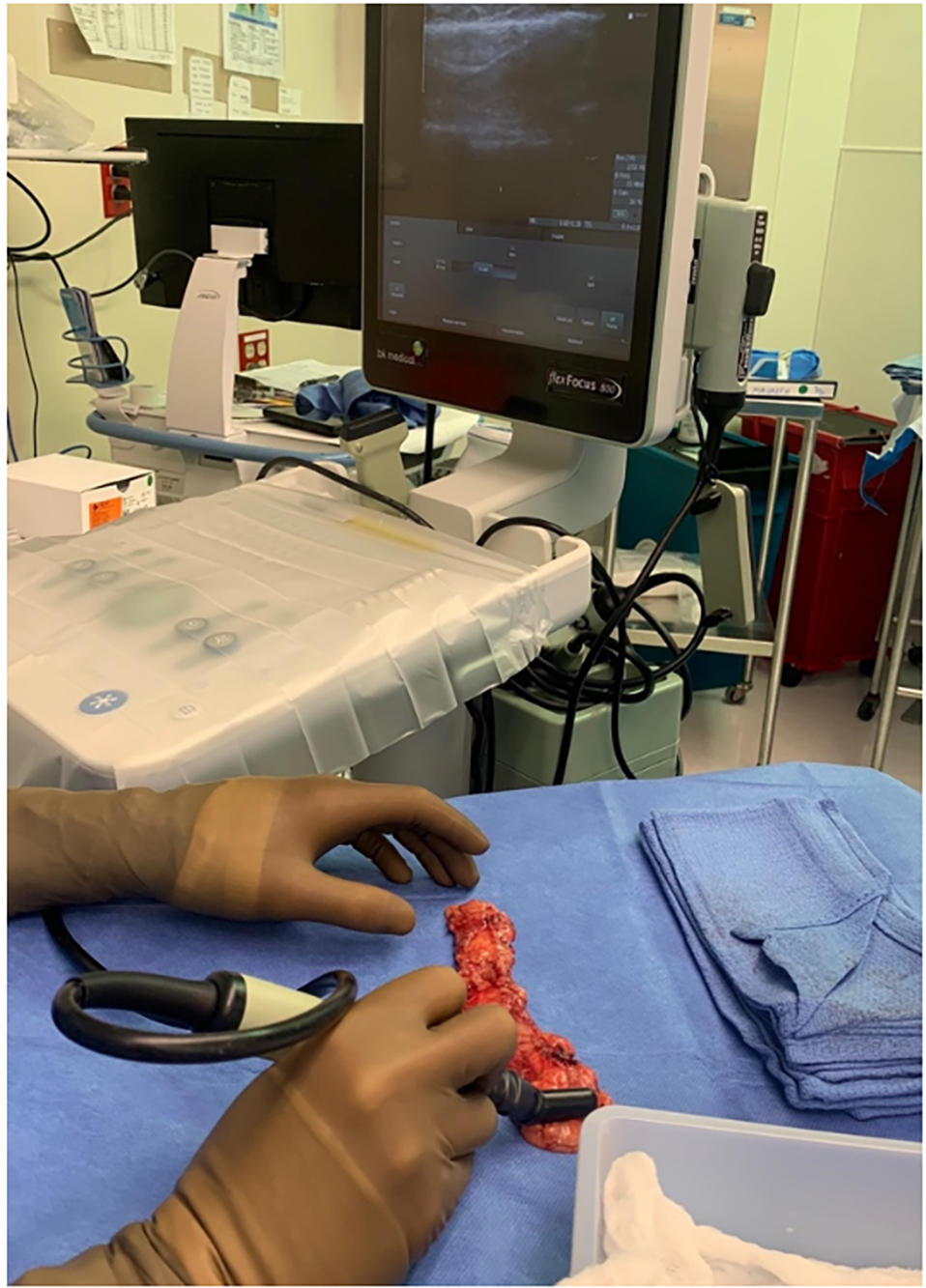
Intraoperative duplex ultrasonography of the gastroepiploic omental lymph node flap for the quantification of transferred lymph nodes.

An attending radiologist (Tsai LL) performed an intraoperative ultrasound on the back table during the gastroepiploic omental harvest [Figure 1]. The number of lymph nodes within the flap and the overall flap weight were recorded. Our intraoperative duplex ultrasound process for lymph node identification and quantification during VLNT has previously been described in detail^[22,23]^.

### Postoperative surveillance

Our standardized process for postoperative surveillance of patients presenting to our Lymphatic Center has previously been described^[18,24]^. Briefly, during postoperative surveillance visits, limb measurements were obtained by a certified lymphedema physical therapist using perometry and L-Dex (Sozo, Impedimed, Carlsbad, California, USA). Relative volume change (RVC) was calculated using the formula, $RVC=\frac{A_{2}\cdot U_{1}}{A_{1}\cdot U_{2}}-1$, where A_1_, U_1_ are the volume of the affected and unaffected limbs prior to VLNT, and A_2_, U_2_ are the volume measurements of the affected and unaffected limbs twelve months post-VLNT^[25]^. Axial fat-suppressed T2-weighted magnetic resonance imaging (MRI) of the affected extremity was obtained at twelve months after debulking in those with fat-dominant disease, as well as twelve months after VLNT in all patients to assess for changes in subcutaneous edema and confirm lymph node flap viability. All MRI studies were read and interpreted by an attending radiologist (Tsai LL).

A validated lymphedema quality-of-life survey (LYMQOL) was administered to patients to assess patient-reported outcomes in four subdomains: appearance, symptoms, mood, and function^[26]^. Patients were queried regarding the number of hours they spent in compression therapy per week and an interval history of any episodes of cellulitis was obtained, which was defined as an infection of the affected extremity requiring treatment with antibiotics.

## RESULTS

During the study period, a total of 23 patients with upper extremity lymphedema were identified, of which 18 met the study inclusion criteria. Five patients were excluded as they had fat-dominant disease and underwent VLNT prior to debulking before we established our current protocol. Of the 18 included, 17 (94%) were female, with a mean age of 57 ± 10 years with a median body mass index of 30 kg/m^[2]^. Seventeen (94%) were identified as Caucasian and one (5%) as Black or African American. The cohort was stratified by fluid-dominance (*n* = 9) and fat-dominance (*n* = 9).

Lymphedema and surgical characteristics were similar among both groups [Table 1]. Of the total cohort, 17 (94%) patients had an oncologic etiology for their lymphedema and one (6%) patient developed lymphedema following an axillary lipoma removal. The time interval from lymphedema diagnosis to the initial surgical consultation appeared to be shorter in the fluid-dominant cohort with a median of 2 (1–4) years compared to the fat-dominant group at 8 (5–15) years. For lymphedema patients with fat-dominant disease, the median time from debulking to VLNT was 18 (16–21) months. For all patients, the median flap weight was 23 (18–28) grams with a median of 6 (5–7) lymph nodes transferred, as identified on intraoperative ultrasound. All lymph node flaps were transferred to the forearm of the affected extremity. Arterial configuration was flow-through^[23]^ in 83% (*n* = 15) of patients and end-to-side in 17% (*n* = 3) of patients. Two venous anastomoses were routinely performed on all flaps. On postoperative day three, one patient developed a right upper extremity hematoma at the operative site, requiring urgent evacuation. No other postoperative complications were reported.

**Figure 2.**
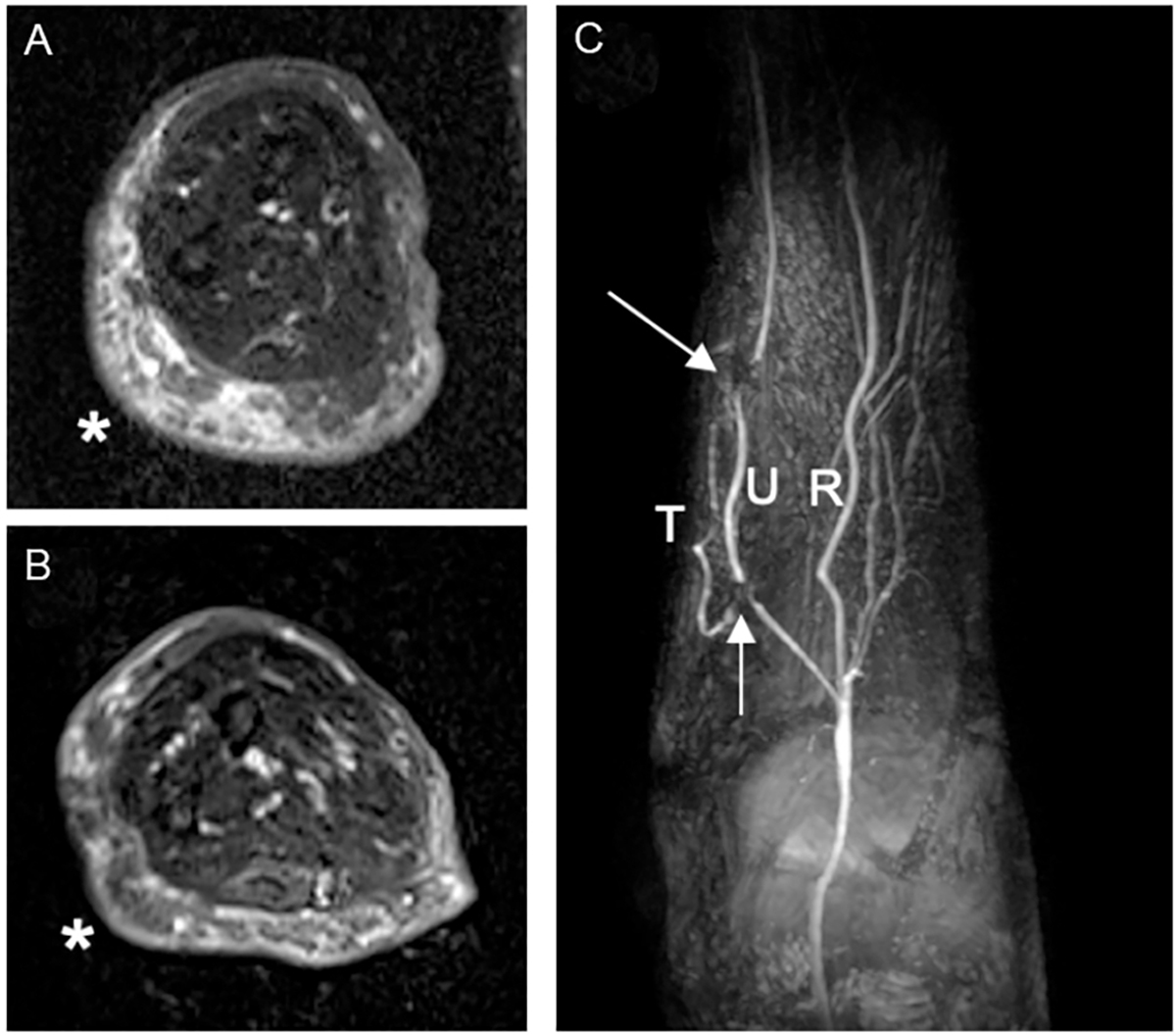
Improvement of upper extremity edema following a vascularized lymph node transplant. Axial fat-suppressed T2-weighted images across the mid right forearm in a patient with right upper extremity lymphedema, pre- (A) and 1-year post-transplant (B) demonstrating interval marked decrease in subcutaneous edema and thickening along the ulnar aspect (*). Arterial-phase post-contrast maximum intensity projection image (C) shows patent flow-through omental artery of the transplant (T). The arrows show signal voids from surgical clips demarcating anastomoses to the ulnar artery (U). R: Radial artery.

At twelve months postoperatively, the fluid-dominant group (*n* = 9) revealed a median limb volume change of −2% (−4% to 2%) with a decrease in hours spent using compression therapy from 47 (1–106) to 4 (0–50) hours per week [Table 2]. This cohort displayed an increase in L-Dex scores from 16 (12–36) to 31 (11–35) and an improvement in all subdomains of LYMQOL at twelve months [Table 3]. Of note, two of the nine patients in this group were able to discontinue compression therapy entirely at twelve months. Of the four patients in this cohort that had postoperative MRI at twelve months, there was a noticeable improvement in edema in 100% (*n* = 4) of patients, and the lymph node flap was viable in 100% (*n* = 4) of the studies [Figure 2].

Overall, the fat-dominant group (*n* = 9) demonstrated a limb volume change of 3% (0%–6%) without a change in the overall hours spent in compression therapy. L-Dex scores remained constant at 17 (4–26) preoperatively and 17 (9–25) at twelve months, and an improvement in all subdomains of LYMQOL was noted, except for the appearance subdomain, which remained unchanged. One of the nine patients in this group was able to discontinue compression therapy entirely at twelve months. Of the eight patients in this cohort that had postoperative MRI at twelve months, there was a noticeable improvement in edema in 75% (*n* = 6) and the lymph node flap was visualized in 88% (*n* = 7) of images.

The median episodes of cellulitis in the fluid-dominant cohort was 1.25 episodes per year preoperatively and 1.05 episodes per year at twelve months postoperatively. The fat-dominant group had a median of 0.3 episodes per year preoperatively and zero episodes per year in the twelve months following VLNT.

## DISCUSSION

In this study, we report our institutional experience and outcomes for omental vascularized lymph node transplant for the treatment of upper extremity lymphedema. Postoperatively, the fluid-dominant cohort demonstrated reductions in both relative limb volume and hours using compression therapy, and had an increase in L-Dex scores at twelve months postoperatively. All LYMQOL subdomain scores improved in this cohort. All patients in this cohort who underwent postoperative imaging revealed an improvement in edema and flap viability on MRI. The fat-dominant cohort had a slight increase in limb volume without an overall change in hours spent using compression therapy or in L-DEX scores, and improvements in quality-of-life scores across almost all subdomains were observed. Of the patients in this cohort who underwent postoperative MRI, 75% displayed an improvement in edema and 88% had confirmed viability of the lymph node flap. Overall, 17% (*n* = 3) of all patients were able to discontinue compression therapy at twelve months postoperatively.

Previous literature has established that VLNT effectively reduces lymphatic fluid accumulation and potentially eliminates the need for compression therapy; however, VLNT does not address the infiltration of fibroadipose tissue^[13,14,27]^. In accordance, our study demonstrated improvements in limb volume measures and a reduction in the hours spent in compression therapy in patients with fluid-dominant lymphedema after undergoing VLNT alone. In the fat-dominant cohort, limb volume had a slight increase without a notable change in compression. We have previously reported that fat-dominant patients undergo significant improvements in limb volume following debulking^[19]^; therefore, we suspect that a ceiling effect may have occurred, indicating that a prior debulking is particularly beneficial for optimizing limb volume in patients with fat-dominant disease. While debulking is targeted at the removal of the fibroadipose tissue, it does not correct the underlying pathophysiologic mechanism of disease, and therefore, patients continue to require lifelong use of compression garments to manage interstitial fluid accumulation^[20,28,29]^. With longer-term follow-up, we anticipate progressively reducing hours in compression while maintaining optimum limb volume in those debulking patients who underwent staged VLNT. Therefore, we propose the sequence of debulking at least one year prior to VLNT for patients with a fat-dominant phenotype to mitigate disease progression and optimize arm volumes prior to VLNT, as VLNT does not typically result in a total reduction of relative limb volume^[8,30,31]^. Moreover, we remain concerned that performing debulking after VLNT may put the transplanted lymph node flap at risk and potentially damage newly formed lymphatic networks^[32–34]^.

Adequate compression therapy has a profound impact on limb volume; thus, it is important to present and interpret changes in limb volume in the context of compression use. In the current study, a reduction in the number of hours spent in compression per week was observed in fluid-dominant patients following VLNT, alongside a reduction in limb volume. The overall hours spent in compression for those with fat-dominant disease was unchanged at one year postoperatively, alongside a minimal increase in limb volume. Three patients in the entire cohort did not require any compression therapy after one year. It is particularly important to report changes in limb volume measurements alongside the time patients spend wearing compression garments. Most prior studies that present patient outcomes following VLNT report compression use as a binary variable (either patients are or are not using compression therapy) or as the percentage of patients able to discontinue compression entirely. Quantifying the extent to which patients use compression garments is valuable, as garments can be burdensome in terms of convenience, time expenditure, cost, and comfort. Additionally, patients presenting to our lymphatic surgery clinic often indicate a decrease in disease management as a treatment goal; therefore, delineating the amount of time spent in compression can help determine whether this goal is being met^[18]^. Additionally, objective measures of limb volume such as RVC and L-Dex can change dramatically over short intervals, so it is useful to interpret these changes in the context of compression garment use. Finally, we note that postoperative changes in limb volume are relatively small in our study. We believe this is closely linked to the fact that our lymphatic surgery program works in tandem with physical therapists in our clinic. Therefore, our patients are already optimized from a limb volume perspective before going to the operating room for VLNT. In this context, hours in compression is an even more important outcome measure.

Improvements in all LYMQOL subdomains were observed in patients with fluid-dominant disease. Similarly, improvements in LYMQOL scores across all subdomains were seen in the fat-dominant group, except for the appearance subdomain, which remained unchanged. The appearance subdomain scores remained relatively constant in patients with fat-dominant disease, possibly because individuals in this cohort likely experienced a dramatic change in their limb volumes following debulking, leading to a major improvement in their perceived appearance that would have occurred prior to VLNT. Overall, the findings from the current study are in concordance with other studies that have reported positive effects of VLNT on patient quality of life^[10,35,36]^. However, in the current study, the beneficial effects may be less directly related to changes in limb volumes and may be more heavily influenced by the reduction in time spent wearing compression garments. Therefore, assessing patient-centered outcomes such as LYMQOL is imperative in gauging whether treatment goals are being met and assessing the efficacy of VLNT procedures.

Despite minimal changes in limb volume and L-Dex scores among both groups at twelve months post-VLNT, MRI studies obtained at this same time point demonstrated noticeable improvement in edema in 83% (*n* = 10) and confirmed lymph node flap viability in 92% (*n* = 11) of patients that underwent postoperative imaging. The radiologic findings in the current study highlight the utility of MRI as an additional modality for measuring subclinical changes in interstitial fluid and additionally underscore the importance of applying a holistic, multi-disciplinary approach for monitoring patients after VLNT. Notably, individual transferred lymph nodes were only visualized on MRI in two patients from the entire cohort, although flap viability was confirmed by MRI in 92%. As the presence and quantity of lymph nodes within the flap were confirmed on intraoperative ultrasound at the time of VLNT, we suspect that our MRIs at the twelve-month time point lack the sensitivity to detect these nodes postoperatively.

A reduction in the median episodes of cellulitis per year was observed in both the fat- and fluid-dominant groups. Only one patient in the fat-dominant cohort had a postoperative case of cellulitis within the twelve months following VLNT, whereas three patients in the fluid-dominant cohort had episodes of cellulitis following VLNT. It is possible that the significantly better outcome that was observed in the fat-dominant patients could be related to the debulking that they previously underwent. This difference may underscore the importance of debulking patients with fat-dominant disease prior to performing VLNT, as debulking targets the removal of fibroadipose tissue, a component that has been established to drive inflammation and clinical progression^[37–39]^. Mitigation of underlying inflammatory processes is likely related to a decrease in postoperative cellulitis occurrences in patients who underwent prior debulking procedures.

This study is not without limitations. While the vast majority of VLNT procedures utilized a flow-through technique for flap anastomosis, in three patients, this technique was not performed. While we believe the flow-through technique is advantageous for enhancing flap hemodynamics^[23]^, it remains uncertain how other techniques used may affect outcomes. Additionally, as data collection was dependent on patient surveillance visits, certain measures were missing from follow-up. Half the study period occurred as we were initiating our center and the second half occurred during the start of the COVID-19 pandemic, during which lymphatic operations and follow-up visits were frequently canceled or rescheduled. This hindered our ability to obtain a complete dataset. Lastly, the sample size was underpowered and data were analyzed descriptively.

Overall, VLNT had varying effects on limb measurements while reliably improving patient quality of life scores. Importantly, VLNT potentially allows patients to reduce or discontinue compression therapy entirely, and in our overall cohort, three patients were able to achieve this goal at twelve months postoperatively. Furthermore, postoperative radiologic improvement in extremity edema and confirmed flap viability were evident among the vast majority of the cohort. Utilizing a staged approach in which debulking is performed prior to VLNT may be particularly useful in alleviating disease in patients with a fat-dominant phenotype, as both fat and fluid components are targeted. This increases the possibility that a patient in this cohort may reduce or discontinue compression therapy, a result that would not have been achieved from debulking alone. This study provides further evidence for VLNT as an effective treatment for lymphedema and underscores the need for consensus on sequence and timing when staging physiologic and debulking procedures for the treatment of lymphedema.

## Figures and Tables

**Table 1.** Patient demographics, disease characteristics, and VLNT intraoperative variables stratified by lymphedema phenotype

	Overall cohort *n* = 18	Fluid-dominant *n* = 9	Fat-dominant *n* = 9

***Baseline characteristics***			
Age at VLNT, yrs (mean ± sd)	57.1 ± 10.1	53.4 ± 9.1	60.8 ± 10.2
Sex, female (*n*, %)	17 (94)	8 (89)	9 (100)
Race (*n*, %)			
White	17 (94)	8 (89)	9 (100)
Black or African American	1 (6)	1 (11)	0 (0)
BMI, kg/m^[2]^ (median, Q1-Q3)	30.2 (28.8–32.1)	30.4 (28.8–32.4)	29.5 (28.5–31.1)
***Lymphedema characteristics***			
Lymphedema laterality (*n*, %)			
Left side	8 (44)	5 (56)	3 (33)
Right side	10 (56)	4 (44)	6 (67)
Limb dominance (*n*, %)			
Left	2 (11)	1 (11)	1 (11)
Right	14 (78)	7 (78)	7 (78)
Ambidextrous	2 (11)	1 (11)	1 (11)
Etiology of lymphedema (*n*, %)			
Oncologic surgery	17 (94)	8 (89)	9 (100)
Non-oncologic surgery	1 (6)	1 (11)	0 (0)
Time from lymphedema diagnosis to VLNT evaluation, years (median, Q1-Q3)	4.2 (2–7.2)	2 (1–4)	8 (4.5–15)
Time from debulking lipectomy to VLNT, months (median, Q1-Q3)	-	-	19.4 (16.1–20.3)
***Surgical characteristics***			
Flap weight, grams (median, Q1-Q3)	23 (17.5–28)	24 (15–29)	22 (18.8–28)
Recipient location (*n*, %)			
Forearm	18 (100)	9 (100)	9 (100)
Flow-through technique utilized, yes (*n*, %)	15 (83)	6 (67)	9 (100)
No. lymph nodes identified by ultrasound (median, Q1-Q3)	6 (5–7)	6 (4–7)	6 (5–7)

VLNT: Vascularized lymph node transplantation.

**Table 2.** Measurements of limb volume and hours spent in compression therapy at the time of preoperative evaluation and 12 months post-VLNT in patients with fat- or fluid-dominant lymphedema phenotypes

	Baseline	12-month visit

**Relative volume change, %**		
Fluid dominant (*n* = 8)		
*Median, Q1-Q3*	-	−2% (−4%–1.6%)
Fat dominant (*n* = 9)		
*Median, Q1-Q3*	-	2.6% (−0.4%–5.6%)
**Compression therapy, hours**		
Fluid dominant (*n* = 6)		
*Median, Q1-Q3*	46.5 (0.8–106)	3.5 (0–49.8)
Fat dominant (*n* = 9)		
*Median, Q1-Q3*	168 (168–168)	168 (168–168)
**L-Dex score**		
Fluid dominant (*n* = 7)		
*Median, Q1-Q3*	15.8 (11.6–36)	30.6 (10.3–34.8)
Fat dominant (*n* = 8)		
*Median, Q1-Q3*	17 (3.6–25.6)	16.8 (8.4–25.4)

VLNT: Vascularized lymph node transplantation.

**Table 3.** LYMQOL Domain Scores at the time of preoperative evaluation and 12 months post-VLNT and at 12 months following VLNT

	Baseline	12-month visit

**Fluid dominant (*n* = 4)**		
Appearance		
*Median, Q1-Q3*	2.6 (1.9–3.3)	1.8 (1.6–2.1)
Functional		
*Median, Q1-Q3*	1.9 (1.6–3.2)	1.4 (1.4–1.8)
Mood		
*Median, Q1-Q3*	2.3 (1.6–3.2)	1.7 (1.6–1.9)
Symptoms		
*Median, Q1-Q3*	2.7 (2.3–2.9)	2.3 (1.8–2.7)
**Fat dominant (*n* = 5)**		
Appearance		
*Median, Q1-Q3*	1.4 (1.4–2.4)	1.4 (1.2–1.6)
Functional		
*Median, Q1-Q3*	1.3 (1.2–1.6)	1.2 (1.1–1.3)
Mood		
*Median, Q1-Q3*	1.7 (1.7–1.8)	1.2 (1.0–1.2)
Symptoms		
*Median, Q1-Q3*	2.0 (1.8–2.2)	1.8 (1.3–2.0)

LYMQOL: Lymphedema quality-of-life; VLNT: vascularized lymph node transplantation.

## Data Availability

Data are inputted and stored in a prospectively maintained REDCap Quality Improvement Database.
